# Evaluation of risk factors for ocular morbidities and its impact on the lives of medical students: An academic collateral.

**DOI:** 10.12688/f1000research.167220.1

**Published:** 2025-11-07

**Authors:** Haneen Haneen, Jarina Begum, Syed Irfan Ali, Abhishek Kumar, Swati Shikha, Khushboo Juneja

**Affiliations:** 1 Manipal Tata Medical College, Manipal Academy of Higher Education, Manipal, 831017, India

**Keywords:** Ocular morbidity, Medical Student, Academics, Lifestyle, Myopia

## Abstract

**Background:**

One billion people worldwide have preventable vision impairment. Ocular morbidities are a significant problem in the public health sector, especially among medical students. The study objectives were to identify the prevailing ocular morbidities and evaluate the risk factors and their impact on students’ lifestyles and academics.

**Methods:**

A cross-sectional study was conducted among 312 undergraduate medical students over 6 months. Data were collected through a structured questionnaire and analysed to identify the prevalence, associated risk factors, and consequences of ocular morbidities.

**Results:**

64.7% were suffering from ocular morbidities. Headache was a predominant symptom in students with (51.7%) and without (39.1%) ocular morbidities. The most common ocular morbidity was myopia (84.3%). 18.7% of students perceived that ocular morbidity had restricted them from participating in activities or applying for specific job posts. The evaluation of various risk factors inferred that ocular morbidity was associated with family history, early age onset of the condition, lighting, posture while reading, screen time, and a vitamin A-rich diet.

**Conclusion:**

The study concluded that the most prevalent ocular morbidity was refractive error, with myopia being the highest among medical students, and it has adversely impacted the students’ lifestyle and academics, underscoring the need for early detection, preventive strategies, and health education interventions.

## Introduction

At least 2.2 billion people have vision impairment worldwide, and among these, 1 billion vision impairments are preventable. ^1^ Avoidable ocular morbidities are a significant public health problem in India. ^2^ Vision is an important and special sense of living beings. These days, it is common to find an increasing number of younger people and even children complaining about several vision problems apart from the age-onset eye conditions. The prevalence of myopia was attributed to urban lifestyle, family history, longer time spent on near-work activities, and fewer outdoor activities. Few surveys have explored the role of high exposure in developing cell phone vision syndrome among college students. ^3^ Environmental factors, such as dryness, potentially contribute to ocular discomfort and alteration of the tear film. ^4^ Ocular morbidities are common among medical students, although many areas in this field require good near and far vision. ^5^ Sleep disorders and underlying diseases are also major concerns pertaining to the onset of vision-related tiredness. ^6^ There is a need for standard vision testing in the young population to diagnose such problems at the earliest. ^7^ The current study aimed to identify the risk factors for various vision problems among medical students that affect their lifestyles.

Medical students are prone to excessive device usage due to recent shifts to online mode during the pandemic, followed by a hybrid mode of study as well as a heavy load of academic involvement. We intend to identify the risk factors for vision problems among such students and their effect on the lifestyle of the students so that the issue of increasing ocular morbidities can be addressed.

### Objectives

1. To identify the ocular morbidities prevailing in medical students.
2. To evaluate the risk factors causing the ocular morbidities.
3. To discuss its impact on the lifestyle & academics of medical students.

## Methodology

### Study design

A cross-sectional study was conducted among 312 undergraduate medical students over six months.

### Sample size

312. The study sample was calculated by using a population proportion of ocular morbidity of 50%, confidence interval (CI) of 95%, population size around 800, and an error of 5%, which is estimated as 260. Assuming a non-response rate of 20%, the sample size was calculated as 312. A sampling method of complete enumeration was used with the inclusion of medical students with ocular morbidities (vision problems) in the age group 18-26 years, currently pursuing their professional course, and who consented to participate in the study. Those lacking any prescription or knowledge of ocular morbidity or those submitting incomplete forms were excluded from the study.

### Study tools

A semi-structured questionnaire was circulated through an online Google survey form, which was developed and validated by a panel of public health experts. The questionnaire comprised five sections of socio-demographic profile, status of ocular morbidity, impact on lifestyle, impact on academics, and open-ended questions discussing the difficulties faced due to ocular morbidity and suggestions to overcome them.

### Ethical approval statement

The study was reviewed and approved by the Manipal Tata Medical College Institutional Ethics Committee (DHR Registration: EC/NEW/INST/2022/2810) with approval number MTMC/IEC/2023/31. All procedures performed involving human participants were in accordance with the ethical standards of the institutional committee and with the 1964 Declaration of Helsinki and its later amendments.

### Data collection

Data were collected using an online Google survey form administered to MBBS students across all phases of study in two medical colleges within the region. Written informed consent was obtained electronically through the survey platform, and only participants who provided consent were permitted to complete the questionnaire. No minors were enrolled in the study. In accordance with the inclusion and exclusion criteria, morbidity details were further gathered through structured telephonic interviews and subsequently verified by subject experts prior to inclusion in the analysis.

### Statistical analysis

Descriptive statistics for quantitative data in the form of percentages and proportions, along with the chi-square test for categorical data, were used. Thematic analysis was performed on qualitative data obtained through open-ended questions.

## Results

Out of 312 majority were females (72.4%) and currently living in hostels (84.4%). Around 83.4% belonged to general caste and high socioeconomic status (86.5%).

202 students (64.7%) were suffering from ocular morbidities currently. Rest of the 110 students (35.3%) had ocular morbidities in the past. However, the students were having ocular symptoms (headache, strain in the eyes, itchiness, watery eyes, eye fatigue, dark circles, dry eye, etc.), among which headaches were the predominant symptom (39.1%). The majority revealed that there were no interventional procedures performed for their past ocular morbidities, except a few (4.3%), which were minor interventions such as chalazion cyst removal, Lasik, trauma, and foreign body in the eye, etc.

Out of 202 students who were suffering from one or other forms of ocular morbidity, the majority had myopia (84.3%), followed by astigmatism (6.4%), hypermetropia (3.3%), strabismus (1.9%), and others (4.1%), including glaucoma, color blindness, retinal thinning, granular corneal dystrophy, and the predominant symptom was headache (51.7%).

The age of onset of the ocular morbidity was ≤15 years in 62.8% of students. The most common presentation was headache (56%), followed by blurred vision (32%) where they were not inability to see the blackboard, red, dry, and watery eyes (8.2%), and regular checkup (3.8%). As perceived by the students, ocular morbidities have been managed; however, the conditions had variable outcomes in terms of 38.9% improvement, 37% not change, 22.7% worsened, and 1.4% were unsure of their conditions.

Likewise, (12.8%) few students had claimed the reason for their development of ocular morbidity after joining medical colleges.

Currently, 89.1% students were using spectacles and rest of them were either contact lenses or both. Amongst them, 88.2% were using it regularly and rest who were not using it regularly. The reasons stated were ignorance, not fashionable, not required for other than academics, non compliance due to unawareness, uncomfortable.

The mean power of right eye and left eye were -1.24 with a lowest of -0.5 to highest of – 8.5 & -1.21 with a lowest of -0.5 to the highest of -6.25 respectively. The majority had the last eye check-up for an average 6.8 months back.

Very few had any interventional procedures (3.8%) or had history (2.9%) majority (91.6%) had a family history of ocular morbidities in their family members. There were no associated co morbidities, except for few (8.6%) such as diabetes, hypertension, thyroid disorder, asthma, migraine, PCOS, epilepsy, bronchitis, and tuberculosis. Only 6% students had mentioned the presence of addiction in their families.

94 students were taking vitamin and mineral supplements attributed to doctors (62.4%) or parent’s (37.6%), including various forms such as Multivitamin, Vitamin B complex, Vitamin D, Iron or IFA, Calcium, Biotin, Omega 3, Vitamin B12, Zinc etc. When asked about the intake of vitamin A-rich food, almost all were eating it consciously or unconsciously in various forms; however, the frequency varied, as shown in Table 1.

**Table 1.** Frequency of vitamin A-rich food intake (N = 312).

SL. No	Frequency of vitamin A rich food intake	Percentage
1	Once a week	43.30%
2	2 or more times a week	30.60%
3	Rarely	13.50%
4	Never	12.60%

Out of the 312, the majority, 84.4% preferred physical books to read, although virtual PDF books were available. Likewise, the majority (67.2%) used tables and chairs while reading, followed by other means, such as bed, couch, floor, and others [ Table 2].

**Table 2.** Posture while reading (N = 312).

Sl. No	Posture while reading	Percentage
1	Sitting on a Chair and table	61.20%
2	Sitting/lying on a couch	22.80%
3	Lying down	9.40%
4	Sitting on floor	4.60%
5	Others	2%

**Figure 1.**
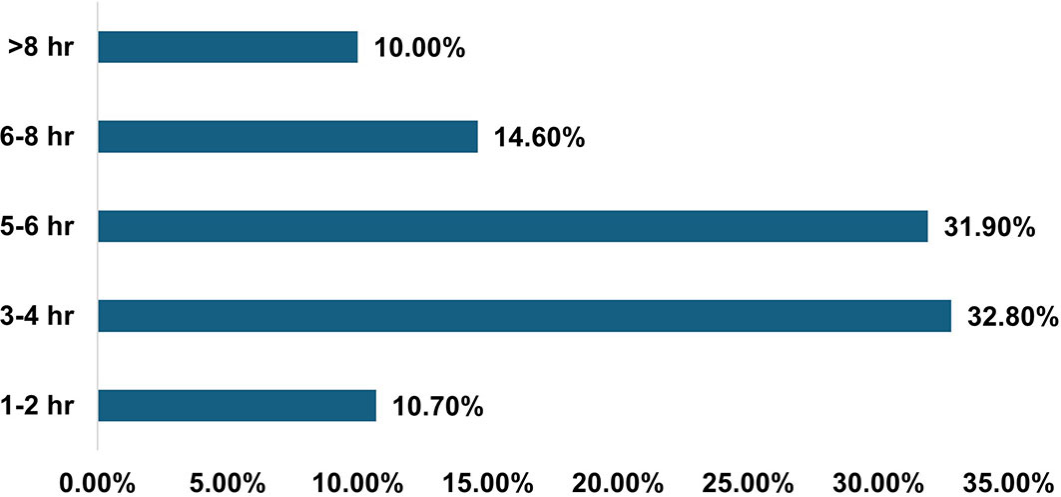
Average screen time of participants (N = 312 Medical Students).

Students had various forms of entertainment, either virtually via electronic devices (59.8%) or physically by interacting with friends and family (58%), followed by outdoor/indoor games (38%), video games, and various other social media interactions. The students were using devices for multiple purposes, but for varied periods of time, as per their needs [ Figure 1].

**Figure 2.**
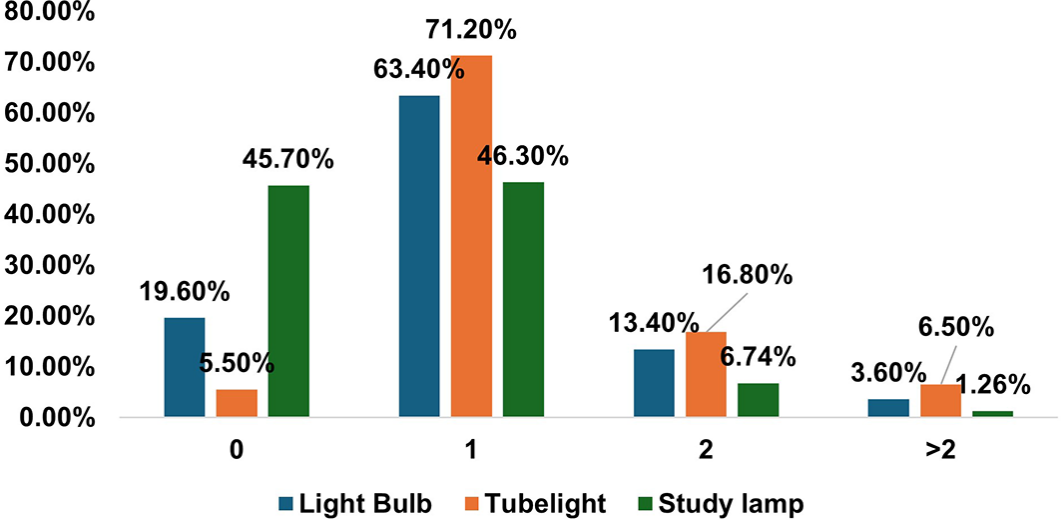
Sources of Artificial Light used by Participants (N = 312 Medical Students).

When asked about adequate lighting, the majority (77.3%) said yes. However, the average number of windows and doors in the reading room was 1.5 and one, respectively, indicating that there was a lack of natural light in the room. Similarly, the average study hours during daylight were less than 3 h compared to study hours without daylight (i.e., 5 h). The majority had a mean sleep hour of 7.3 in the night and a total of 8.6 hours during the 24hours of the day and night. The sources of artificial lights were either light bulbs, tube lights, study lamps, or a combination of them [ Figure 2].

Early age of onset (<15 years of age) and regular eye check-ups (at least once in 6 months) were associated with improved outcomes (P < 0.0001) with respect to ocular morbid conditions. Inappropriate posture, more than five hours of screen time, and device intimacy were significantly associated (P < 0.0001) with ocular morbidities. The association between perceived adequacy of lighting and the occurrence of morbid ocular conditions was found to be insignificant. (P = 0.7705). Female sex and family history were also associated with ocular morbidities (P < 0.0001). Multivitamin supplementation was associated with a lower incidence of any form of ocular morbidity (P < 0.0001) [ Table 3].

**Table 3.** Association of various risk factors with ocular morbidity (N = 312).

Risk factors		Yes	No	P value
**1. Family History**	**Present**	192	93	p < 0.0001
	**Absent**	10	17	
**2. Screen Time**	>5 hr	144	33	p < 0.0001
	<5-hr	58	77	
**3. Device intimacy**	Present	142	44	p < 0.0001
	Absent	60	66	
**4.Gender**	Female	162	63	p < 0.0001
	Male	40	47	
**5. Light**	Adequate	155	86	P = 0.7705
	Inadequate	47	24	
**6. Posture**	Right	104	86	p < 0.0001
	Wrong	98	24	
**7. Vitamin Supplementation**	Present	24	70	p < 0.0001
	Absent	178	40	
		**Improved or No change**	**Worsened**	
**8. Age of onset**	During school (<_15 yr)	61	66	p < 0.0001
	After school (>15 Years)	59	16	
**9. Routine check-ups **	<6 months	102	47	p < 0.0001
	>6 months	18	35	

18.7% of students confessed that ocular morbidity had restricted them from participating in activities or applying for a post, such as swimming, military/army/air force post, dance, a few sports, basketball, badminton, cricket, driving at night, watching 3D movies, continuous screen use, and seeing through microscopes, etc.

**Figure 3.**
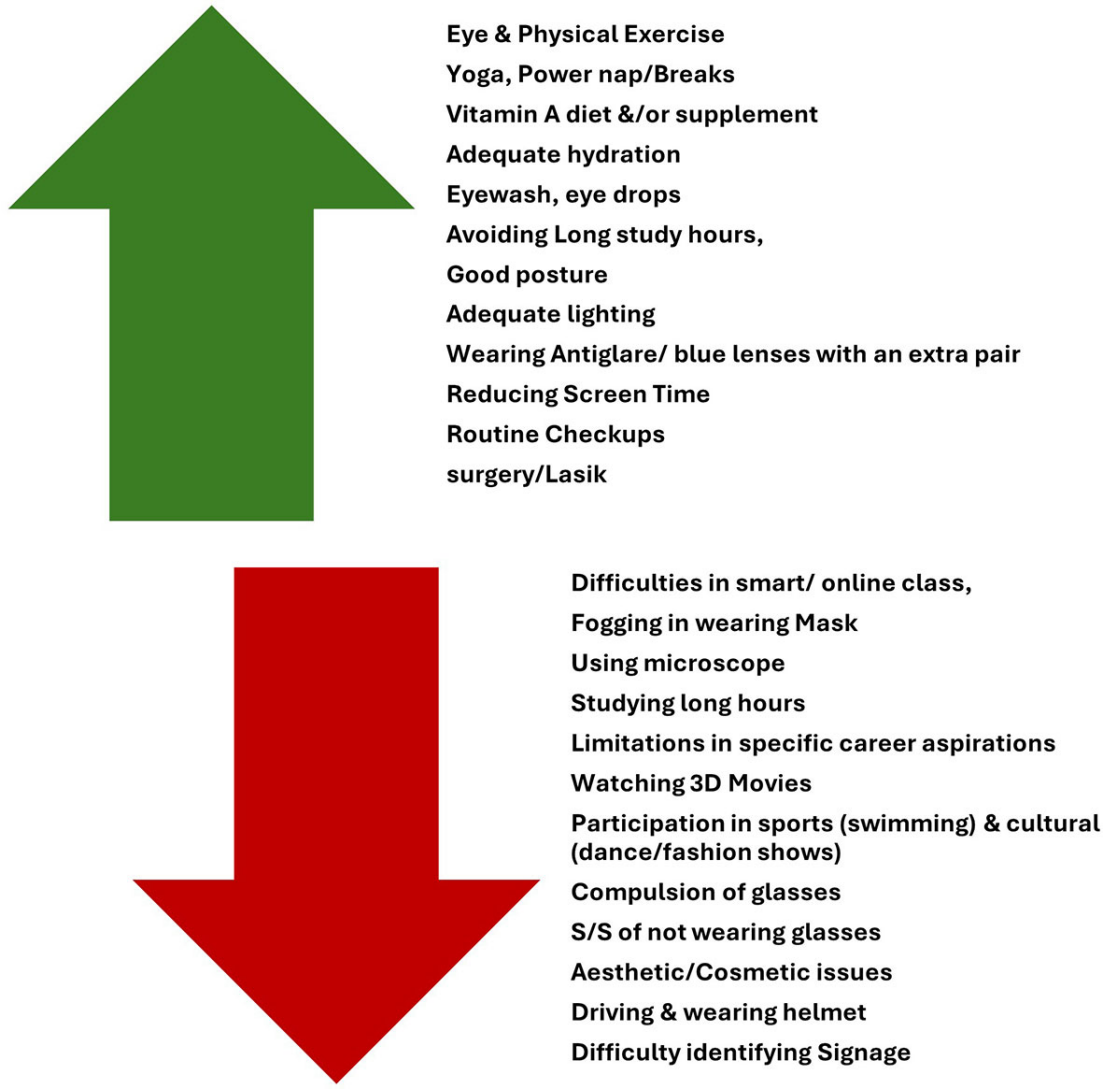
Challenges and suggested solutions for ocular morbidities.

The students mentioned various challenges encountered with ocular morbidity and suggested solutions to overcome them through open-ended questions, which were presented as themes [ Figure 3].

## Discussion

The majority (57.4%) had myopia in the current study followed by other conditions. Studies has stated Refractive error is the most common ocular morbid condition among children. ^8^ Likewise the most common presentation of ocular symptoms at onset was blurred vision, followed by headache. However, another study found that headache (50%) and dry eyes (45%) were the common ocular complaints, and myopia was common as well among the students. ^9^

The students were concerned about the challenges encountered when dealing with ocular morbidities, such as the inability to study long hours and engaging with devices. Likewise, an observational study was conducted among 200 medical students who are using smart phone, laptops, and computers to determine the relationship between eye strain and related risk factors, which showed that most of the students had more than one symptom, such as headache (56.77%), eye strain (50.52%), blurring of vision (40.62%), and redness (23.95%). 85% of patients used electronic devices for longer duration of 4-10 hours, had more asthenopia or eye strain. ^10^

Although inappropriate posture, more than five hours of screen time, and device intimacy had a significant association, the perceived adequacy of lighting had no statistically significant association with the occurrence of ocular morbid conditions. Another study on the prevalence of eye strain and other ocular morbidities among students inferred that low levels of lighting in the classroom may be an contributing factor for ocular morbidity. ^11^

The current study observed that female students and those with a family history of ocular morbidity were more vulnerable to ocular morbidity. Another study showed similar results, with a higher prevalence of ocular morbidity among females. ^8^

The students suggested solutions to the perceived challenges pertaining to ocular morbidities, such as a vitamin A-rich diet, reduced screen time, yoga, hydration, and regular eye check-ups. Similarly, age <17years was found to be another factor responsible for the high prevalence of ocular morbidities, to which the suggested reasonable services were early screening and health education. Another study among medical students observed common ocular morbidities, such as refractive errors, dry eyes, and color blindness, directing frequent eye checkups. ^12, 13^

As discussed in the study, students were predominantly myopic and using spectacles; they also perceived limitations in playing outdoor games and sports due to their ocular morbidity. Studies have found similar findings where myopia was the common ocular morbidity among medical students as a huge portion of their time was spent on near –work and doing indoor activities while very less in doing outdoor activities, which was indicated as a risk factor for myopia. ^14, 15^

## Conclusion

The study concluded that the most prevalent ocular morbidity among medical students was refractive error, with myopia being the most prevalent. Evaluation of various risk factors inferred that ocular morbidity in students was associated with familial history, female sex, appropriate posture while reading, screen time, device intimacy, and multivitamin supplementation. Ocular morbid conditions have adversely affected the students’ lifestyles and academics in terms of their inability to participate in sports and games, career aspirations, such as in the Army or Air Force, Navy, etc. Likewise, the inability to study long hours is another negative effect of ocular morbidity as perceived by the students, underscoring the need for early detection, preventive strategies, and health education interventions.

### Limitations

A small sample size and an online questionnaire were used for data collection. There is scope for a larger study in the future to explore ocular morbidity among healthcare professionals.

## Data Availability

The datasets generated and analysed during the current study are not publicly available due to the inclusion of sensitive participant information. However, they are available from the corresponding author upon reasonable request (Email: jarina.begum@manipal.edu). Access will be granted for legitimate research purposes, provided that appropriate ethical approvals are obtained, and the confidentiality of participants can be assured.
