# Evaluation of risk factors for ocular morbidities and their impact on the lives of medical students: A cross-sectional study unveiling the academic collateral.

**DOI:** 10.12688/f1000research.167220.2

**Published:** 2026-01-10

**Authors:** Haneen Haneen, Jarina Begum, Syed Irfan Ali, Abhishek Kumar, Swati Shikha, Khushboo Juneja

**Affiliations:** 1 Manipal Tata Medical College, Manipal Academy of Higher Education, Manipal, 831017, India

**Keywords:** Ocular morbidity, Medical Student, Academics, Lifestyle, Myopia

## Abstract

**Background:**

One billion people worldwide have preventable vision impairment. Ocular morbidities are a significant problem in the public health sector, especially among medical students. The study objectives were to identify the prevailing ocular morbidities and evaluate the risk factors and their impact on students’ lifestyles and academics.

**Methods:**

A cross-sectional study was conducted among (Study sample 312) undergraduate medical students over 6 months. Data were collected through a structured questionnaire and analysed to identify the prevalence, associated risk factors, and consequences of ocular morbidities.

**Results:**

64.7% were suffering from ocular morbidities. Headache was a predominant symptom in students with (51.7%) and without (39.1%) ocular morbidities. The most common ocular morbidity was myopia (84.3%). 18.7% of students perceived that ocular morbidity had restricted them from participating in activities or applying for specific job posts. The evaluation of various risk factors inferred that ocular morbidity was associated with family history, early age onset of the condition, lighting, inappropriate posture while reading, screen time, and a vitamin A-rich diet.

**Conclusion:**

The study concluded that the most prevalent ocular morbidity was refractive error, with myopia being the highest among medical students, and it has adversely impacted the students’ lifestyle and academics, underscoring the need for early detection, preventive strategies, and health education interventions.

## Introduction

At least 2.2 billion people have vision impairment worldwide, and among these, 1 billion vision impairments are preventable. ^1^ Avoidable ocular morbidities are a significant public health problem in India. ^2^ Vision is an important and special sense of living beings. These days, it is common to find an increasing number of younger people and even children complaining about several vision problems apart from the age-onset eye conditions. The prevalence of myopia was attributed to urban lifestyle, family history, longer time spent on near-work activities, and fewer outdoor activities. Few surveys have explored the role of high exposure in developing cell phone vision syndrome among college students. ^3^ Environmental factors, such as dryness, potentially contribute to ocular discomfort and alteration of the tear film. ^4^ Ocular morbidities are common among medical students, although many areas in this field require good near and far vision. ^5^ Sleep disorders and underlying diseases are also major concerns pertaining to the onset of vision-related tiredness. ^6^ There is a need for standard vision testing in the young population to diagnose such problems at the earliest. ^7^ The current study aimed to identify the risk factors for various vision problems among medical students that affect their lifestyles.

There are **unique academic and occupational visual demands** faced by medical students, including prolonged microscope use during practical sessions, extensive reading requirements, sustained digital screen exposure, and irregular schedules with late-night studying and clinical duties. These factors collectively distinguish medical students from other university populations and provide a strong justification for their focused evaluation in relation to ocular morbidities.

Medical students are prone to excessive device usage due to recent shifts to online mode during the pandemic, followed by a hybrid mode of study as well as a heavy load of academic involvement. We intend to identify the risk factors for vision problems among such students and their effect on the lifestyle of the students so that the issue of increasing ocular morbidities can be addressed.

### Objectives

1. To identify the prevailing ocular morbidities in medical students.
2. To evaluate the risk factors causing the ocular morbidities.
3. To discuss its impact on the lifestyle & academics of medical students.

## Methodology

The study was conducted among undergraduate MBBS students enrolled across all academic phases in two medical colleges in the study area, with an approximate total student population of 800. The study was not restricted to students with ocular complaints.

An attempted complete enumeration (census-based) sampling approach was adopted, whereby all eligible medical students were invited to participate irrespective of their ocular health status, until the required sample size of 312 was achieved. No sampling frame, random selection, or restriction based on ocular complaints was applied. An online questionnaire link was circulated through official institutional communication channels, inviting voluntary participation. This approach ensured representation across all academic phases and allowed estimation of the prevalence and pattern of ocular morbidities within the study population.

Undergraduate medical students aged 18–26 years who were currently enrolled in the MBBS course and provided informed consent were included. Students who submitted incomplete or partially filled questionnaires or were unable to provide reliable information regarding their ocular health status were excluded from the analysis.

### Study design

Descriptive analytical cross-sectional study.

### Sample size

312. The study sample was calculated by using a population proportion of ocular morbidity of 50%, confidence interval (CI) of 95%, population size around 800, and an error of 5%, which is estimated as 260. Assuming a non-response rate of 20%, the sample size was calculated as 312.

### Study tools

A semi-structured questionnaire was circulated through an online Google survey form, which was developed and validated by a panel of public health experts. The questionnaire comprised five sections of socio-demographic profile, status of ocular morbidity, impact on lifestyle, impact on academics, and open-ended questions discussing the difficulties faced due to ocular morbidity and suggestions to overcome them.

Intake of vitamin A–rich foods was assessed using a self-reported frequency-based measure. Participants were asked to report their usual intake of commonly consumed vitamin A–rich food items listed in the questionnaire, with response options categorized as never, rarely, once per week, or two or more times per week.

Lighting conditions and posture during reading and screen use were assessed using self-reported structured questions in the questionnaire. Participants were asked to indicate the primary source(s) of lighting used during reading or screen-related activities (light bulb, tube light, study lamp), including the number of light sources used (none, one, two, or more than two). Reading posture was assessed by asking participants to report their usual posture while studying, with predefined response options including sitting at a chair and table, sitting or lying on a couch, lying down, sitting on the floor, or other postures.

For analytical purposes, daily screen time was dichotomized as <5 hours and ≥5 hours, based on existing literature indicating increased ocular strain with prolonged screen exposure beyond 4–5 hours per day.

These variables were included to explore ergonomic and environmental factors potentially associated with ocular morbidity. As these variables were self-reported, they reflect participants’ usual practices and perceptions and were not objectively measured.

The impact of ocular morbidities on lifestyle and academic activities was assessed using a dedicated section of the semi-structured questionnaire. Academic impact was evaluated through self-reported difficulties related to educational activities, including challenges in prolonged reading, extended screen use, viewing classroom teaching aids (such as blackboard or projected slides), and use of microscopes during practical sessions.

Lifestyle impact was assessed by documenting self-reported restrictions in routine and recreational activities, including participation in sports, swimming, night-time driving, and perceived limitations related to future career aspirations (e.g., eligibility for armed forces or other visually demanding professions).

In addition to closed-ended items, open-ended questions were included to capture participants’ perceived challenges related to ocular morbidity and the coping strategies or solutions adopted. Responses to open-ended questions were analysed using thematic analysis to identify recurring patterns and themes.

### Ethical approval statement

The study was reviewed and approved by the Manipal Tata Medical College Institutional Ethics Committee (DHR Registration: EC/NEW/INST/2022/2810) with approval number MTMC/IEC/2023/31. All procedures performed involving human participants were in accordance with the ethical standards of the institutional committee and with the 1964 Declaration of Helsinki and its later amendments.

### Data collection

Data were collected using an online Google survey form administered to MBBS students across all phases of study in two medical colleges within the region. Participation was entirely voluntary. Students were provided with detailed study information through the online survey platform and were invited to indicate their willingness to participate by providing electronic informed consent. Only those who voluntarily chose to consent proceeded to complete the questionnaire and enrol in the study.

This was a questionnaire-based cross-sectional study, and no direct clinical ocular examinations (including refraction, slit-lamp examination, or fundoscopy) were performed as part of the study protocol. Information on ocular morbidities—such as refractive errors (myopia, hypermetropia, astigmatism), strabismus, glaucoma, dry eye disease, allergic conjunctivitis, and other reported ocular conditions—was obtained through self-reported history of prior diagnosis, use of corrective measures, and available prescriptions.

For participants reporting ocular morbidities, additional details were collected through structured telephonic interviews. Where feasible, these self-reported details were cross-verified with available medical records or prescriptions and reviewed by subject experts prior to inclusion in the final analysis, to enhance data accuracy and reproducibility.

Ocular morbidity status was classified based on self-reported presence or absence of a current ocular condition at the time of the survey, thereby representing point prevalence rather than lifetime prevalence.

### Statistical analysis

Descriptive statistics for quantitative data in the form of percentages and proportions, along with the chi-square test for categorical data, were used. Thematic analysis was performed on qualitative data obtained through open-ended questions.

## Results

Out of 312 majority were females (72.4%) and currently living in hostels (84.4%). Around 83.4% belonged to general caste and high socioeconomic status (86.5%).

At the time of the survey, 202 students (64.7%) reported having a current ocular morbidity, while 110 students (35.3%) reported no current ocular morbidity. The students with ocular morbidity were having ocular symptoms (headache, strain in the eyes, itchiness, watery eyes, eye fatigue, dark circles, dry eye, etc.), among which headaches were the predominant symptom (39.1%). The majority revealed that there were no interventional procedures performed in their past, except a few (4.3%), which were minor interventions such as chalazion cyst removal, Lasik, trauma, and foreign body in the eye, etc.

Out of 202 students who were suffering from one or other forms of ocular morbidity, the majority had myopia (84.3%), followed by astigmatism (6.4%), hypermetropia (3.3%), strabismus (1.9%), and others (4.1%), including glaucoma, color blindness, retinal thinning, granular corneal dystrophy, and the predominant symptom was headache (51.7%).

The age of onset of the ocular morbidity was ≤15 years in 62.8% of students. The most common presenting complaint was headache (56%), followed by blurred vision (32%), including difficulty seeing the blackboard, redness, dryness, and watering of eyes (8.2%), and routine eye check-ups (3.8%). As perceived by the students, ocular morbidities have been managed; however, the conditions had variable outcomes in terms of 38.9% improvement, 37% not change, 22.7% worsened, and 1.4% were unsure of their conditions.

12.8% of students perceived that the onset of their ocular morbidity occurred after joining medical college. This observation is based on self-reported perception and does not imply a causal relationship or objective evidence.

Currently, 89.1% students were using spectacles and rest of them were either contact lenses or both. Amongst them, 88.2% were using it regularly and rest who were not using it regularly. The reasons stated were ignorance, not fashionable, not required for other than academics, non compliance due to unawareness, uncomfortable.

The mean power of right eye and left eye were -1.24 with a lowest of -0.5 to highest of – 8.5 & -1.21 with a lowest of -0.5 to the highest of -6.25 respectively. The majority had the last eye check-up for an average 6.8 months back.

Very few had any interventional procedures (3.8%) or had history (2.9%) majority (91.6%) had a family history of ocular morbidities in their family members. There were no associated co morbidities, except for few (8.6%) such as diabetes, hypertension, thyroid disorder, asthma, migraine, PCOS, epilepsy, bronchitis, and tuberculosis. Only 6% students had mentioned the presence of addiction in their families.

94 students were taking vitamin and mineral supplements attributed to doctors (62.4%) or parent’s (37.6%), including various forms such as Multivitamin, Vitamin B complex, Vitamin D, Iron or IFA, Calcium, Biotin, Omega 3, Vitamin B12, Zinc etc. When asked about the intake of vitamin A-rich food, almost all were eating it consciously or unconsciously in various forms; however, the frequency varied, as shown in Table 1.

**Table 1.** Frequency of vitamin A-rich food intake (N = 312).

SL. No	Frequency of vitamin A rich food intake	Percentage
1	Once a week	43.30%
2	2 or more times a week	30.60%
3	Rarely	13.50%
4	Never	12.60%

Out of the 312, the majority, 84.4% preferred physical books to read, although virtual PDF books were available. Likewise, the majority (67.2%) used tables and chairs while reading, followed by other means, such as bed, couch, floor, and others [ Table 2].

**Table 2.** Posture while reading (N = 312).

Sl. No	Posture while reading	Percentage
1	Sitting on a Chair and table	61.20%
2	Sitting/lying on a couch	22.80%
3	Lying down	9.40%
4	Sitting on floor	4.60%
5	Others	2%

**Figure 1.**
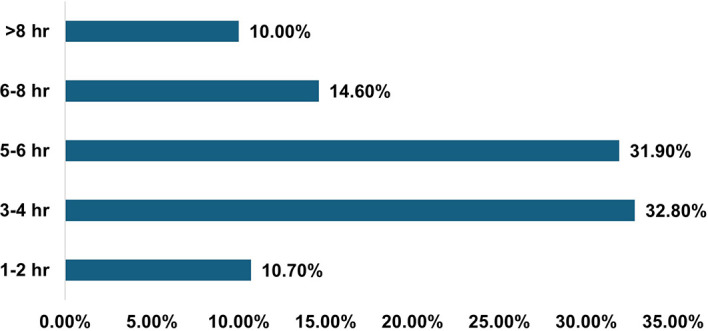
Average screen time of participants (N = 312 Medical Students).

Students had various forms of entertainment, either virtually via electronic devices (59.8%) or physically by interacting with friends and family (58%), followed by outdoor/indoor games (38%), video games, and various other social media interactions. The students were using devices for multiple purposes, but for varied periods of time, as per their needs [ Figure 1].

**Figure 2.**
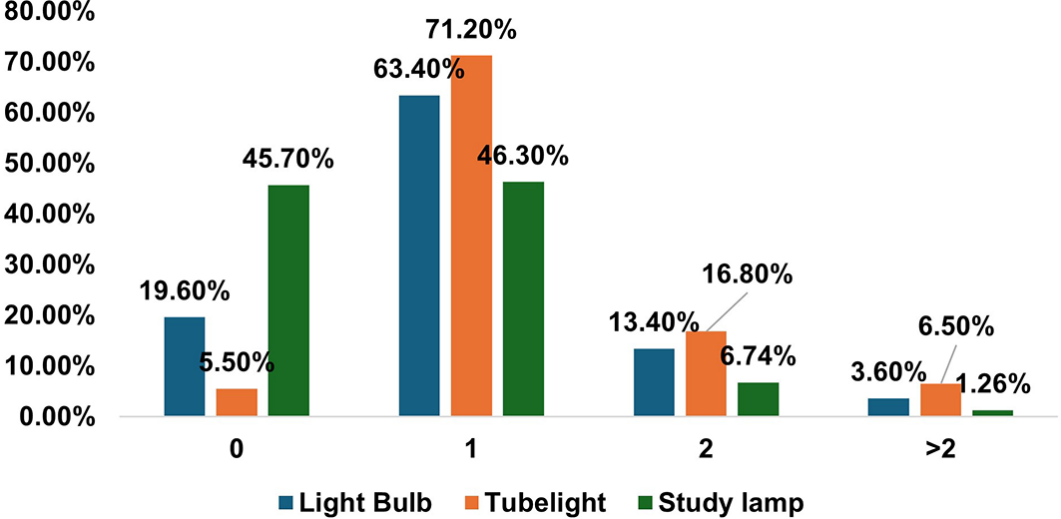
Sources of Artificial Light used by Participants (N = 312 Medical Students).

When asked about adequate lighting, the majority (77.3%) said yes. However, the average number of windows and doors in the reading room was 1.5 and one, respectively, indicating that there was a lack of natural light in the room. Similarly, the average study hours during daylight were less than 3 h compared to study hours without daylight (i.e., 5 h). The majority had a mean sleep hour of 7.3 in the night and a total of 8.6 hours during the 24hours of the day and night. The sources of artificial lights were either light bulbs, tube lights, study lamps, or a combination of them [ Figure 2].

Early age of onset (<15 years of age) and regular eye check-ups (at least once in 6 months) were associated with improved outcomes (P < 0.0001) with respect to ocular morbid conditions. Inappropriate posture, more than five hours of screen time, and device intimacy were significantly associated (P < 0.0001) with ocular morbidities. The association between perceived adequacy of lighting and the occurrence of morbid ocular conditions was found to be insignificant. (P = 0.7705). Female sex and family history were also associated with ocular morbidities (P < 0.0001). However, the statistically significant association observed may, in part, be an artefact of the sample composition rather than a definitive biological or behavioral relationship attributed to female overrepresentation in the study participants. Multivitamin supplementation was associated with a lower incidence of any form of ocular morbidity (P < 0.0001) [ Table 3].

**Table 3.** Association of various risk factors with ocular morbidity (N = 312).

Risk factors		Yes	No	P value
**1. Family History**	**Present**	192	93	p < 0.0001
	**Absent**	10	17	
**2. Screen Time**	>5 hr	144	33	p < 0.0001
	<5-hr	58	77	
**3. Device intimacy**	Present	142	44	p < 0.0001
	Absent	60	66	
**4. Gender**	Female	162	63	p < 0.0001
	Male	40	47	
**5. Light**	Adequate	155	86	P = 0.7705
	Inadequate	47	24	
**6. Posture**	Right	104	86	p < 0.0001
	Wrong	98	24	
**7. Vitamin Supplementation**	Present	24	70	p < 0.0001
	Absent	178	40	

Among students with ocular morbidity, earlier age of onset (≤15 years) was more frequently associated with worsening of symptoms compared to later onset (>15 years) ( *p* < 0.0001). Students who had undergone an eye examination within the past six months more commonly reported improvement or no change, whereas worsening was more frequent among those with longer intervals since their last check-up ( *p* < 0.0001).

18.7% of students confessed that ocular morbidity had restricted them from participating in activities or applying for a post, such as swimming, military/army/air force post, dance, a few sports, basketball, badminton, cricket, driving at night, watching 3D movies, continuous screen use, and seeing through microscopes, etc.

**Figure 3.**
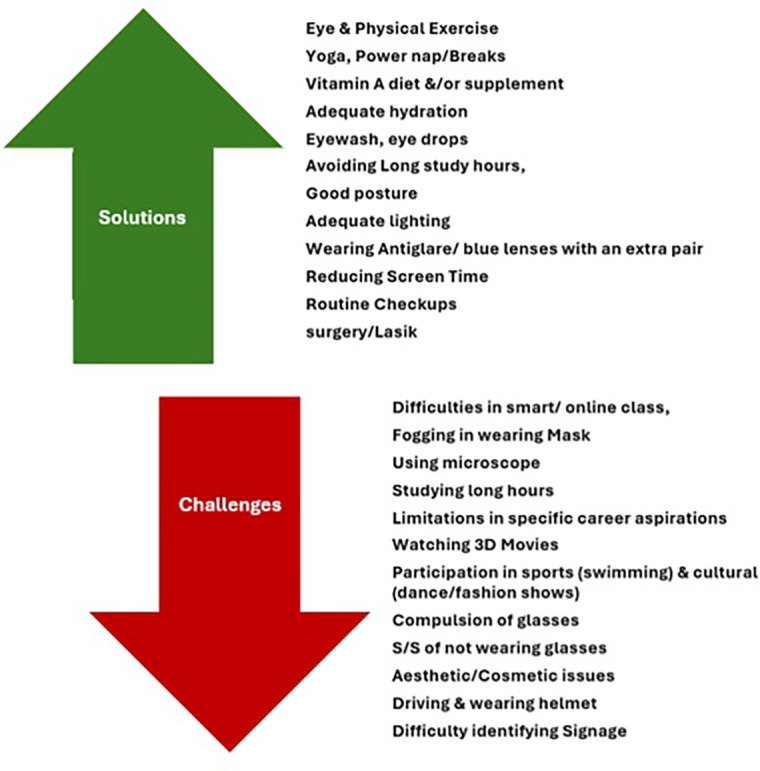
Self-reported challenges and coping strategies related to ocular morbidities among medical students.

The students mentioned various challenges encountered with ocular morbidity and suggested solutions to overcome them through open-ended questions, which were presented as themes [ Figure 3].

## Discussion

This study describes the spectrum of ocular morbidities among undergraduate medical students and explores associated risk factors and perceived impacts on lifestyle and academic activities. Among the 202 students with current ocular morbidity, refractive errors were predominant, with myopia accounting for 84.3% of cases. Overall, myopia was present in 57.4% of the total study population (n = 312), being the most common condition. Similar patterns have been reported in previous studies among school-going children and medical students, where refractive errors constituted the major burden of ocular morbidity. ^8^ The high prevalence of myopia among medical students may reflect sustained near-work demands and limited outdoor activities inherent to medical training.

Blurred vision and headache were the most frequently reported symptoms at the onset associated with ocular morbidity in the present study. Comparable findings have been reported among medical students and young adults, where headache, eye strain, dry eye and visual fatigue were common presenting complaints, particularly in the context of prolonged screen exposure. ^9^^,^^10^ These symptoms are consistent with asthenopic manifestations frequently observed in populations with high digital device use.

Several ergonomic and behavioral factors were explored as potential risk factors. Prolonged screen time, inappropriate reading posture, and close viewing distances were commonly reported among students with ocular morbidity. These findings align with earlier observational studies demonstrating an association between extended digital device use and symptoms of eye strain among medical students. It also showed that most of the students had more than one symptom, such as headache (56.77%), eye strain (50.52%), blurring of vision (40.62%), & redness (23.95%). Moreover, 85% of patients used electronic devices for a longer duration of 4-10 hours, and had more asthenopia or eye strain. ^10^

While perceived adequacy of lighting was assessed, there was no statistically significant association; no definitive inference regarding its role could be made, as lighting conditions were self-reported and not objectively measured. Nevertheless, previous studies have suggested that inadequate classroom illumination may contribute to ocular discomfort and eye strain, highlighting the importance of optimal lighting in learning environments. ^11^

Female students and those reporting a family history of ocular morbidity appeared to have a higher prevalence of ocular conditions in this study. Similar trends have been documented in other studies, suggesting that genetic predisposition and gender-related behavioral or biological factors may influence ocular morbidity patterns. ^8^ However, given the cross-sectional design, these observations should be interpreted as associations rather than causal relationships.

A subset of students perceived that their ocular morbidity developed after joining medical college. This perception likely reflects increased visual demands and lifestyle changes during medical training; however, this finding is based solely on self-report and does not establish temporal or causal relationships. The study design does not permit attribution of ocular morbidity onset to medical education itself.

The study findings indicate that incorrect or ergonomically suboptimal reading posture is associated with a higher prevalence of ocular morbidity, underscoring the importance of ergonomic education and posture correction as potential preventive strategies.

Beyond clinical patterns, students highlighted several perceived challenges related to ocular morbidity, including difficulty studying for prolonged hours, discomfort during online classes, limitations in sports and extracurricular activities, aesthetic concerns related to spectacle use, and practical difficulties such as fogging while wearing masks. These findings underscore the broader lifestyle and academic implications of ocular morbidity, which are often underrepresented in quantitative assessments. Mehta et al. similarly noted that myopia among medical students was associated with reduced participation in outdoor activities, which may further exacerbate visual strain and progression of refractive errors. ^12^

Participants also reported various self-adopted coping strategies, including reduced screen time, use of appropriate lighting, maintaining proper posture, regular eye check-ups, hydration, yoga, and consumption of vitamin A–rich foods. While these strategies reflect awareness and adaptive behavior, their effectiveness was not objectively evaluated in this study. Similar recommendations emphasizing early screening, visual hygiene, and preventive practices have been highlighted in other studies involving student populations. ^13^^,^^14^ Comparable preventive recommendations, including periodic ophthalmic screening and visual hygiene practices, have been emphasized by Rizyal et al. ^15^

It is important to note that the impact described in this study reflects **students’ perceptions and experiences**, rather than objectively measured academic performance or functional outcomes. The inclusion of open-ended responses allowed students to articulate individualized challenges and adaptive strategies, highlighting the broader psychosocial and lifestyle implications of ocular morbidity beyond clinical diagnosis alone.

Overall, the findings indicate that ocular morbidities among medical students are common, multifactorial, and associated with perceived academic and lifestyle challenges. The consistency of these findings with studies from diverse geographic settings underscores the need for institutional strategies such as regular vision screening, ergonomic education, and promotion of visual hygiene practices within medical colleges. Longitudinal studies incorporating objective ophthalmic assessments and detailed exposure measurement would help clarify causal pathways and inform targeted preventive interventions.

## Conclusion

This study found that refractive errors were the most prevalent ocular morbidities among medical students, with myopia being the most common. Several factors were **associated** with the presence of ocular morbidity, including family history of ocular conditions, female sex, inappropriate reading posture, prolonged screen time, close device viewing distance, and multivitamin supplementation.

Ocular morbidities were perceived by students to have a negative impact on both lifestyle and academic activities, particularly through reduced participation in sports and recreational activities, perceived limitations in career aspirations requiring optimal visual acuity (such as the Armed Forces), and difficulty sustaining prolonged periods of study. These findings highlight the need for early identification of ocular morbidities, promotion of ergonomic practices and visual hygiene, and implementation of preventive and health education interventions targeted at medical students.

### Limitations

A small sample size and an online questionnaire were used for data collection. As participation was voluntary, non-response occurred, and the potential for selection bias, incomplete participation resulted in a respondent-based sample, which may limit generalizability of prevalence estimates.

Reliance on self-reported data, and the inherent limitations of a cross-sectional study design.

A major limitation of this study is the female predominance among respondents (72.4%), which may reflect differential response behavior rather than the true gender distribution of the source population. This imbalance may have influenced the observed association between female sex and ocular morbidity; therefore, gender-based findings should be interpreted cautiously and not generalized beyond the study population.

Screen time was dichotomized for analytical purposes to facilitate interpretation; however, this approach may have resulted in the loss of information inherent to the original multi-category variable, which is acknowledged as one of the study’s limitations.

There is scope for a larger study in the future to explore ocular morbidity among healthcare professionals and the need for future studies with balanced gender representation or stratified sampling to more accurately assess sex-related differences in ocular morbidity among medical students.

## Data Availability

The datasets generated and analysed during the current study are not publicly available due to the inclusion of sensitive participant information. However, they are available from the corresponding author upon reasonable request (Email: jarina.begum@manipal.edu). Access will be granted for legitimate research purposes, provided that appropriate ethical approvals are obtained, and the confidentiality of participants can be assured.
