# Supplementary material for: Evaluation of risk factors for ocular morbidities and their impact on the lives of medical students: A cross-sectional study unveiling the academic collateral
Source: F1000Res. 2026 Jan 10;14:1230. Originally published 2025 Nov 7. [Version 2] doi: 10.12688/f1000research.167220.2 (PMC12820473; doi:10.12688/f1000research.167220.2)
Supplement: Peer review history

# Reviewer report: Reviewer response for version 2

**DOI:** 10.5256/f1000research.194747.r449134

**Published:** 2026-01-20

**Authors:** Akinsola S Aina

**Affiliations:** 1 Bowen University, Iwo, Nigeria

My initial queries were already addressed in this revised version of the manuscript.

 It is now fit for indexing.

Is the work clearly and accurately presented and does it cite the current literature?

Partly

If applicable, is the statistical analysis and its interpretation appropriate?

Partly

Are all the source data underlying the results available to ensure full reproducibility?

Yes

Is the study design appropriate and is the work technically sound?

Yes

Are the conclusions drawn adequately supported by the results?

No

Are sufficient details of methods and analysis provided to allow replication by others?

No

Reviewer Expertise:

Ophthalmology(Cornea and Anterior Segment; Medical Retina)

I confirm that I have read this submission and believe that I have an appropriate level of expertise to confirm that it is of an acceptable scientific standard.

# Reviewer report: Reviewer response for version 1

**DOI:** 10.5256/f1000research.184311.r431126

**Published:** 2025-12-24

**Authors:** Samudyatha U chandrashekara

**Affiliations:** 1 Sri Devaraj URS Medical College, Kolar, Karnataka, India

The study, conducted among medical students, highlight the health seeking of medical students for ocular problems. The following suggestions may be noted:

 1. The objectives in the the abstract state that the study aims to estimate the prevalence. However, this differs from the manuscript

 2. Inclusion criteria may be revisited to clarify whether all students were included or those with ocular comorbidity were included. If only those with ocular morbidity were included, then please clarify the objectives.

 3. "Written informed consent was obtained electronically through the survey platform, and only participants who provided consent were permitted to complete the questionnaire." : Please reframe this so that the patient autonomy and right to participate is vested in the participant, and not in the researcher.

 4. "Likewise, (12.8%) few students had claimed the reason for their development of ocular morbidity after joining medical colleges." This statement must be clearly presented as the participant's perceived belief, since it does not have any linked evidence.

 5" When asked about the intake of vitamin A-rich food, ": Please elaborate in methods how this was assessed.

 6. Please include how lighting, posture etc was assessed in the methodology

 7. Figure 3 is interesting. Please indicate which arrow indicate challenge and solution. Also, please indicate what the arrows represent - hierarchy/ time? Consider a simple representation if possible

 8. Kindly reframe the discussion based on the changes in objective and methods

Is the work clearly and accurately presented and does it cite the current literature?

Yes

If applicable, is the statistical analysis and its interpretation appropriate?

Yes

Are all the source data underlying the results available to ensure full reproducibility?

Partly

Is the study design appropriate and is the work technically sound?

Partly

Are the conclusions drawn adequately supported by the results?

Partly

Are sufficient details of methods and analysis provided to allow replication by others?

Partly

Reviewer Expertise:

Infectious disease epidemiology, Health education

I confirm that I have read this submission and believe that I have an appropriate level of expertise to confirm that it is of an acceptable scientific standard, however I have significant reservations, as outlined above.

# Response

**Published:** 2026-01-02

**Authors:** Jarina Begum

**Affiliations:** Community Medicine, Manipal Academy of Higher Education, Jamshedpur, Jharkhand, India

We, the authors of this article, sincerely thank the reviewer for the careful and constructive evaluation of our manuscript titled **“Evaluation of risk factors for ocular morbidities and its impact on the lives of medical students”**

 The comments have been extremely valuable in improving the clarity, methodological rigor, and interpretability of our work. We address each point below.

 1. The objectives in the the abstract state that the study aims to estimate the prevalence. However, this differs from the manuscript.

**Response**- We thank the reviewer for highlighting this inconsistency in the use of words. The objectives in the abstract have now been aligned precisely with the objectives stated in the main manuscript. i.e. To identify the prevailing ocular morbidities in medical students.

 2. Inclusion criteria may be revisited to clarify whether all students were included or those with ocular comorbidity were included. If only those with ocular morbidity were included, then please clarify the objectives.

**Response**- We agree that this required clarification. The Methods section has been revised to explicitly state that the study was carried out among undergraduate MBBS students enrolled across all academic phases in a medical college, with an approximate total student population of 800. and the sample size is estimated as 312. ****A complete enumeration approach was employed, wherein all eligible medical students were invited to participate until the required sample size was achieved. This approach ensured representation from all academic phases. Those undergraduate medical students aged 18–26 years, currently enrolled in the MBBS course, and who provided informed consent to participate were included in the study, and students with incomplete or partially filled questionnaires, unable to provide reliable information regarding ocular health status, were excluded. This brings clarity to the objectives section regarding the inclusion criteria.

 3. "Written informed consent was obtained electronically through the survey platform, and only participants who provided consent were permitted to complete the questionnaire." : Please reframe this so that the patient autonomy and right to participate is vested in the participant, and not in the researcher.

**Response-** Participation was entirely voluntary. Students were provided with detailed study information through the online survey platform and were invited to indicate their willingness to participate by providing electronic informed consent. Only those who voluntarily chose to consent proceeded to complete the questionnaire.

 4. "Likewise, (12.8%) few students had claimed the reason for their development of ocular morbidity after joining medical colleges." This statement must be clearly presented as the participant's perceived belief, since it does not have any linked evidence.

**Response**-We agree. This statement in result section has now been rephrased as-approximately 12.8% of students perceived that the onset of their ocular morbidity occurred after joining medical college. This observation is based on self-reported perception and does not imply a causal relationship or objective evidence.

 5" When asked about the intake of vitamin A-rich food, ": Please elaborate in methods how this was assessed.

**Response: **The Methods section has been expanded to specify that intake of vitamin A–rich foods was assessed using a self-reported frequency-based measure. Participants were asked to report their usual intake of commonly consumed vitamin A–rich food items listed in the questionnaire, with response options categorized as never, rarely, once per week, or two or more times per week.

 6. Please include how lighting, posture, etc., was assessed in the methodology.

**Response-** We thank the reviewer for this suggestion. The Methods section has been revised to clearly describe the assessment of lighting conditions and reading posture using self-reported, structured questionnaire items with predefined response categories, thereby improving methodological transparency and reproducibility. Lighting conditions and posture during reading and screen use were assessed using self-reported structured questions in the questionnaire. Participants were asked to indicate the primary source(s) of lighting used during reading or screen-related activities (light bulb, tube light, study lamp), including the number of light sources used (none, one, two, or more than two). Reading posture was assessed by asking participants to report their usual posture while studying, with predefined response options including sitting at a chair and table, sitting or lying on a couch, lying down, sitting on the floor, or other postures. These variables were included to explore ergonomic and environmental factors potentially associated with ocular morbidity. As these variables were self-reported, they reflect participants’ usual practices and perceptions and were not objectively measured.

 7. Figure 3 is interesting. Please indicate which arrow indicate challenge and solution. Also, please indicate what the arrows represent - hierarchy/ time? Consider a simple representation if possible

**Response-** We thank the reviewer for this constructive suggestion. Figure 3 has been revised to clearly label the upward arrow as self-reported solutions and the downward arrow as self-reported challenges related to ocular morbidity. The arrows serve as conceptual groupings and do not indicate hierarchy or time. The figure has been simplified and accompanied by a clear legend for improved clarity. (Legend- Figure 3. Self-reported challenges and coping strategies related to ocular morbidities among medical students.)

 8. Kindly reframe the discussion based on the changes in objective and methods

**Response- **The discussion section has been rephrased as per the suggestions.

 Discussion- This study describes the spectrum of ocular morbidities among undergraduate medical students and explores associated risk factors and perceived impacts on lifestyle and academic activities. Refractive errors emerged as the predominant ocular morbidity, with myopia (57.4%) being the most common condition. Similar patterns have been reported in previous studies among school-going children and medical students, where refractive errors constituted the major burden of ocular morbidity.⁸ The high prevalence of myopia among medical students may reflect sustained near-work demands and limited outdoor activities inherent to medical training.

 Blurred vision and headache were the most frequently reported symptoms at the onset associated with ocular morbidity in the present study. Comparable findings have been reported among medical students and young adults, where headache, eye strain, dry eye and visual fatigue were common presenting complaints, particularly in the context of prolonged screen exposure.⁹,¹⁰ These symptoms are consistent with asthenopic manifestations frequently observed in populations with high digital device use.

 Several ergonomic and behavioral factors were explored as potential risk factors. Prolonged screen time, inappropriate reading posture, and close viewing distances were commonly reported among students with ocular morbidity. These findings align with earlier observational studies demonstrating an association between extended digital device use and symptoms of eye strain among medical students. It also showed that most of the students had more than one symptom, such as headache (56.77%), eye strain

 (50.52%), blurring of vision (40.62%), and redness (23.95%). Moreover, 85% of patients used electronic devices for a longer duration of 4-10 hours, and had more asthenopia or eye strain.¹⁰

 While perceived adequacy of lighting was assessed, there was no statistically significant association; no definitive inference regarding its role could be made, as lighting conditions were self-reported and not objectively measured. Nevertheless, previous studies have suggested that inadequate classroom illumination may contribute to ocular discomfort and eye strain, highlighting the importance of optimal lighting in learning environments.¹¹

 Female students and those reporting a family history of ocular morbidity appeared to have a higher prevalence of ocular conditions in this study. Similar trends have been documented in other studies, suggesting that genetic predisposition and gender-related behavioral or biological factors may influence ocular morbidity patterns.⁸ However, given the cross-sectional design, these observations should be interpreted as associations rather than causal relationships.

 A subset of students perceived that their ocular morbidity developed after joining medical college. This perception likely reflects increased visual demands and lifestyle changes during medical training; however, this finding is based solely on self-report and does not establish temporal or causal relationships. The study design does not permit attribution of ocular morbidity onset to medical education itself.

 Beyond clinical patterns, students highlighted several perceived challenges related to ocular morbidity, including difficulty studying for prolonged hours, discomfort during online classes, limitations in sports and extracurricular activities, aesthetic concerns related to spectacle use, and practical difficulties such as fogging while wearing masks. These findings underscore the broader lifestyle and academic implications of ocular morbidity, which are often underrepresented in quantitative assessments. Mehta et al. similarly noted that myopia among medical students was associated with reduced participation in outdoor activities, which may further exacerbate visual strain and progression of refractive errors. ^12^

 Participants also reported various self-adopted coping strategies, including reduced screen time, use of appropriate lighting, maintaining proper posture, regular eye check-ups, hydration, yoga, and consumption of vitamin A–rich foods. While these strategies reflect awareness and adaptive behavior, their effectiveness was not objectively evaluated in this study. Similar recommendations emphasizing early screening, visual hygiene, and preventive practices have been highlighted in other studies involving student populations. ^13, 14^ Comparable preventive recommendations, including periodic ophthalmic screening and visual hygiene practices, have been emphasized by Rizyal et al. ^15^

 Overall, the findings indicate that ocular morbidities among medical students are common, multifactorial, and associated with perceived academic and lifestyle challenges. The consistency of these findings with studies from diverse geographic settings underscores the need for institutional strategies such as regular vision screening, ergonomic education, and promotion of visual hygiene practices within medical colleges. Longitudinal studies incorporating objective ophthalmic assessments and detailed exposure measurement would help clarify causal pathways and inform targeted preventive interventions.

 The revised references written as-

 12. Mehta R, Bedi N, Punjabi S: Prevalence of myopia in medical

 students. Indian Journal of Clinical and Experimental Ophthalmology. 2019; 5(3): 322–325.

 Publisher Full Text

 13. Rao GN, Sabnam S, Pal S, et al. : Prevalence of ocular morbidity among children aged 17 years or younger in the eastern India. Clinical ophthalmology (Auckland, N.Z.). 2018; Volume 12: 1645–1652. PubMed Abstract|Publisher Full Text|Free Full Text

 14. Shrestha P, Kaiti R, Shyangbo R, et al.: Ocular Survey in Kathmandu University Medical Students. Kathmandu University Medical Journal (KUMJ). 2022 Apr-Jun; 20(78): 209–213.

 PubMed Abstract|Publisher Full Text

 15. Rizyal A, Shrestha RK, Mishal A, et al. : Ocular disorders and

 associated factors among the first year health professional

 students at a medical college in Kathmandu. Nepal Med. Coll. J.

 2022; 24(3): 206–212.Publisher Full Text

# Reviewer report: Reviewer response for version 1

**DOI:** 10.5256/f1000research.184311.r433453

**Published:** 2025-12-24

**Authors:** Raya Khudhair Mohsin

**Affiliations:** 1 Nursing college, Al-Farabi University, Baghdad, Iraq

Manuscript Title: Evaluation of risk factors for ocular morbidities and its impact on the lives of medical students: An academic collateral

 Manuscript ID/DOI: 10.12688/f1000research.167220.1

 Authors: Haneen Haneen, Jarina Begum, Syed Irfan Ali, Abhishek Kumar, Swati Shikha, Khushboo Juneja

 Journal: F1000Research

 Review Date: 12/04/2025

 Overall Recommendation: Major Revision

 The manuscript addresses a relevant and timely topic—ocular health among medical students—which is of significant public health and educational interest. The study employs a mixed-methods approach, collecting both quantitative and qualitative data, which adds depth to the findings. However, several methodological, analytical, and presentational issues currently limit the validity, clarity, and impact of the work. Substantive revisions are required before the manuscript can be considered suitable for publication.

 General Comments

 The research question is pertinent, especially in the context of increasing digital device use in medical education. The inclusion of student-suggested solutions is a strength, as it bridges the gap between identification of problems and potential interventions. However, the manuscript in its current form suffers from critical flaws in the description of the methodology, inconsistencies in data reporting, misinterpretation of results, and an underdeveloped discussion of limitations.

 Major Issues

 1. Methodological Clarity and Sampling Bias:

    · "Complete Enumeration" Misnomer: The methodology states that "a sampling method of complete enumeration was used." However, a sample of 312 from a population of 800 is a sample, not a census. This term is incorrect and misleading. The exact recruitment strategy must be transparently described (e.g., Was it a voluntary online survey sent to all students? Was it a random sample?). The current description suggests a convenience or voluntary response sampling method, which carries a high risk of selection bias. Individuals with eye problems may have been more motivated to participate, potentially inflating prevalence estimates.

    · Severe Sample Demographics Skew: The sample is 72.4% female. This extreme gender imbalance is a critical limitation that is not addressed. It likely biases key results, most notably the reported significant association between female sex and ocular morbidity (P < 0.0001). This finding may be an artifact of the sample composition rather than a true biological or behavioral association. This must be highlighted as a major limitation and caution must be applied when generalizing the gender-based finding.

 2. Results: Inconsistencies and Misinterpretation:

    · Prevalence Confusion: The Abstract and Results sections present conflicting data. The Abstract states: "64.7% were suffering from ocular morbidities." The Results section (Page 4) states: "202 students (64.7%) were suffering from ocular morbidities currently. Rest of the 110 students (35.3%) had ocular morbidities in the past." This implies a 100% lifetime prevalence, which is confusing and biologically improbable. The text must clearly differentiate between point prevalence (current) and lifetime prevalence, and the logic of this categorization should be explained.

    · Critical Error in Conclusion: The Abstract and Conclusion state that "appropriate posture while reading" is associated with ocular morbidity. This is a direct misinterpretation of the data in Table 3, which clearly shows that "Wrong" posture is significantly associated with morbidity (P < 0.0001). The manuscript must be corrected to state that inappropriate posture is a risk factor. This error fundamentally changes the public health message of the study.

 3. Data Analysis and Presentation:

    · Unjustified Data Dichotomization: Figure 1 presents screen time in multiple categories (1-2 hrs, 3-4 hrs, etc.). However, for analysis in Table 3, screen time is dichotomized into >5 hrs vs. <5 hrs. The rationale for choosing this specific cutoff point is not provided. This arbitrary dichotomization loses information and should be justified, orHere is a comprehensive peer review report for the manuscript, structured according to standard academic review guidelines

 Manuscript Title: Evaluation of risk factors for ocular morbidities and its impact on the lives of medical students: An academic collateral

 Manuscript ID/DOI: 10.12688/f1000research.167220.1

 Authors: Haneen Haneen, Jarina Begum, Syed Irfan Ali, Abhishek Kumar, Swati Shikha, Khushboo Juneja

 Journal: F1000Research

 Review Date: 12/02/2025

 Overall Recommendation: Major Revision

 The manuscript addresses a relevant and timely topic—ocular health among medical students—which is of significant public health and educational interest. The study employs a mixed-methods approach, collecting both quantitative and qualitative data, which adds depth to the findings. However, several methodological, analytical, and presentational issues currently limit the validity, clarity, and impact of the work. Substantive revisions are required before the manuscript can be considered suitable for publication.

 General Comments

 The research question is pertinent, especially in the context of increasing digital device use in medical education. The inclusion of student-suggested solutions is a strength, as it bridges the gap between identification of problems and potential interventions. However, the manuscript in its current form suffers from critical flaws in the description of the methodology, inconsistencies in data reporting, misinterpretation of results, and an underdeveloped discussion of limitations.

 Major Issues

 1. Methodological Clarity and Sampling Bias:

    · "Complete Enumeration" Misnomer: The methodology states that "a sampling method of complete enumeration was used." However, a sample of 312 from a population of 800 is a sample, not a census. This term is incorrect and misleading. The exact recruitment strategy must be transparently described (e.g., Was it a voluntary online survey sent to all students? Was it a random sample?). The current description suggests a convenience or voluntary response sampling method, which carries a high risk of selection bias. Individuals with eye problems may have been more motivated to participate, potentially inflating prevalence estimates.

    · Severe Sample Demographics Skew: The sample is 72.4% female. This extreme gender imbalance is a critical limitation that is not addressed. It likely biases key results, most notably the reported significant association between female sex and ocular morbidity (P < 0.0001). This finding may be an artifact of the sample composition rather than a true biological or behavioral association. This must be highlighted as a major limitation and caution must be applied when generalizing the gender-based finding.

 2. Results: Inconsistencies and Misinterpretation:

    · Prevalence Confusion: The Abstract and Results sections present conflicting data. The Abstract states: "64.7% were suffering from ocular morbidities." The Results section (Page 4) states: "202 students (64.7%) were suffering from ocular morbidities currently. Rest of the 110 students (35.3%) had ocular morbidities in the past." This implies a 100% lifetime prevalence, which is confusing and biologically improbable. The text must clearly differentiate between point prevalence (current) and lifetime prevalence, and the logic of this categorization should be explained.

    · Critical Error in Conclusion: The Abstract and Conclusion state that "appropriate posture while reading" is associated with ocular morbidity. This is a direct misinterpretation of the data in Table 3, which clearly shows that "Wrong" posture is significantly associated with morbidity (P < 0.0001). The manuscript must be corrected to state that inappropriate posture is a risk factor. This error fundamentally changes the public health message of the study.

 3. Data Analysis and Presentation:

    · Unjustified Data Dichotomization: Figure 1 presents screen time in multiple categories (1-2 hrs, 3-4 hrs, etc.). However, for analysis in Table 3, screen time is dichotomized into >5 hrs vs. <5 hrs. The rationale for choosing this specific cutoff point is not provided. this arbitrary dichotomization loses information and should be justified,or analysis using the original categories should be considered.

    · Confusing Table 3 Format: Table 3 is poorly constructed and difficult to interpret. The column headers are inconsistent. For risk factors 1-7, the columns are "Yes" and "No," but for factors 8 and 9 (Age of onset, Routine check-ups), the columns shift to "Improved or No change" and "Worsened." This creates confusion about what is being compared. The table should be redesigned for consistency and clarity, ideally comparing the presence/absence of ocular morbidity across all risk factor categories.

    · Figure 3 (Challenges and Solutions): While informative, this figure is cluttered and difficult to read. It is recommended to split this into two separate, clear lists or a structured table to improve readability.

 4. Underdeveloped Limitations Section:

    · The current limitations mention only "a small sample size" and "an online questionnaire." This is insufficient. The section must be expanded to explicitly discuss:

      · The non-probability sampling method and high risk of selection bias.

      · The severe gender imbalance in the sample and its implications.

      · Reliance on self-reported data for both morbidity and risk factors (susceptible to recall and social desirability bias).

      · The cross-sectional design, which precludes any causal inferences. The language throughout the manuscript should be tempered from "causing" or "effects" to "associated with."

 Minor Issues

 · Title: The subtitle "An academic collateral" is unclear. Suggest a more standard and descriptive subtitle such as "A cross-sectional study."

 · Introduction: The rationale for specifically studying medical students (vs. other university students) could be strengthened by emphasizing unique stressors like prolonged microscope use, vast reading loads, and night-duty schedules.

 · Author List & Roles (Page 2): There is a discrepancy. The author list includes "Swati Shikha," but the "Author roles" section lists "Kimura A" and "Jungla K." These names ("Kimura A," "Jungla K") do not appear in the main author list and are likely errors that need correction to "Swati Shikha" and "Khushboo Juneja."

 · Results Text (Page 4): The phrasing "where they were not inability to see the blackboard" appears to be a grammatical error or incomplete sentence.

 · Discussion: The first sentence states "The majority (57.4%) had myopia," but the Results on Page 4 state the prevalence among those with morbidity is 84.3%. Please verify and use consistent statistics.

 · References: Reference #2 appears to be cut off or corrupted: "(POP) Prevalence of Smart Phone Users at Risk...". This needs to be checked and formatted correctly.

 Summary of Required Revisions

 1. Methodology: Rewrite the sampling section to accurately describe the recruitment process and replace "complete enumeration" with the correct term. Acknowledge the limitations of the sampling method.

 2. Results: Clarify the prevalence definitions (current vs. past). Correct the major error regarding "posture" in the Abstract and Conclusion. Redesign Table 3 for clarity and consistency. Justify the screen time cutoff.

 3. Analysis & Limitations: Conduct a sensitivity analysis or explicitly discuss the impact of the gender imbalance on the results. Significantly expand the Limitations section to address sampling bias, gender skew, self-report bias, and the cross-sectional design.

 4. Presentation: Correct author name errors. Split or reformat Figure 3 for clarity. Perform a thorough proofread for grammar and consistency.

 5. Discussion: Ensure all data cited from the results are accurate. Temper language to reflect associational, not causal, findings.

 Potential Impact

 Once the major methodological and interpretive issues are addressed, this study has the potential to contribute valuable insights into the ocular health challenges faced by medical students. The qualitative findings regarding perceived impact and student-suggested solutions are particularly useful for designing targeted health promotion interventions within medical colleges.

 The reviewer appreciates the opport unity to assess this work and is available to review a revised manuscript.

 Best regards,

 [Dr.Raya Khudhair]

Is the work clearly and accurately presented and does it cite the current literature?

Yes

If applicable, is the statistical analysis and its interpretation appropriate?

Partly

Are all the source data underlying the results available to ensure full reproducibility?

Yes

Is the study design appropriate and is the work technically sound?

Partly

Are the conclusions drawn adequately supported by the results?

Yes

Are sufficient details of methods and analysis provided to allow replication by others?

Yes

Reviewer Expertise:

general practictioner (family medicine)

I confirm that I have read this submission and believe that I have an appropriate level of expertise to confirm that it is of an acceptable scientific standard, however I have significant reservations, as outlined above.

# Response

**Published:** 2026-01-02

**Authors:** Jarina Begum

**Affiliations:** Community Medicine, Manipal Academy of Higher Education, Jamshedpur, Jharkhand, India

We as the authors of this article sincerely thank Dr. Raya Khudhair for the detailed, thoughtful, and constructive review, which has been invaluable in improving the methodological clarity, analytical rigor, and overall quality of our manuscript; all comments have been carefully addressed, as detailed in the point-by-point responses below

 Major Issues

 1. Methodological Clarity and Sampling Bias:

    · "Complete Enumeration" Misnomer: The methodology states that "a sampling method of complete enumeration was used." However, a sample of 312 from a population of 800 is a sample, not a census. This term is incorrect and misleading. The exact recruitment strategy must be transparently described (e.g., Was it a voluntary online survey sent to all students? Was it a random sample?). The current description suggests a convenience or voluntary response sampling method, which carries a high risk of selection bias. Individuals with eye problems may have been more motivated to participate, potentially inflating prevalence estimates.

**Response- **We thank the reviewer for this important methodological comment. We agree that the final analyzed sample represents a subset of the total eligible population. However, we would like to clarify that the **sampling strategy was an attempted complete enumeration (census-based approach)** rather than sample-based selection. We acknowledge that voluntary non-response may introduce selection bias, which has now been explicitly stated as a limitation. The Methods section has been revised to clarify that this was an **attempted complete enumeration with respondent-based analysis**, thereby avoiding ambiguity and improving transparency.

 Methodology Reframed as- The study was conducted among undergraduate MBBS students enrolled across all academic phases in two medical colleges in the study area, with an approximate total student population of 800. The study was not restricted to students with ocular complaints.

 An attempted complete enumeration (census-based) sampling approach was adopted, whereby all eligible medical students were invited to participate irrespective of their ocular health status, until the required sample size of 312 was achieved. No sampling frame, random selection, or restriction based on ocular complaints was applied. An online questionnaire link was circulated through official institutional communication channels, inviting voluntary participation. This approach ensured representation across all academic phases and allowed estimation of the prevalence and pattern of ocular morbidities within the study population.

 Undergraduate medical students aged 18–26 years who were currently enrolled in the MBBS course and provided informed consent were included. Students who submitted incomplete or partially filled questionnaires or were unable to provide reliable information regarding their ocular health status were excluded from the analysis

 Limitations rephrased as- A small sample size and an online questionnaire were used for data collection. As participation was voluntary, non-response occurred, and the potential for selection bias, incomplete participation resulted in a respondent-based sample, which may limit generalizability of prevalence estimates.

 Reliance on self-reported data, and the inherent limitations of a cross-sectional study design.

 A major limitation of this study is the female predominance among respondents (72.4%), which may reflect differential response behavior rather than the true gender distribution of the source population. This imbalance may have influenced the observed association between female sex and ocular morbidity; therefore, gender-based findings should be interpreted cautiously and not generalized beyond the study population.

 Screen time was dichotomized for analytical purposes to facilitate interpretation; however, this approach may have resulted in loss of information inherent to the original multi-category variable, which is acknowledged as one of the study limitation.

 There is scope for a larger study in the future to explore ocular morbidity among healthcare professionals and the need for future studies with balanced gender representation or stratified sampling to more accurately assess sex-related differences in ocular morbidity among medical students.

 Severe Sample Demographics Skew: The sample is 72.4% female. This extreme gender imbalance is a critical limitation that is not addressed. It likely biases key results, most notably the reported significant association between female sex and ocular morbidity (P < 0.0001). This finding may be an artifact of the sample composition rather than a true biological or behavioral association. This must be highlighted as a major limitation and caution must be applied when generalizing the gender-based finding.

**Response:** We thank the reviewer for this important and valid observation. We acknowledge that the study sample demonstrated a marked female predominance (72.4%), which reflects the gender distribution among respondents rather than an intentionally balanced sampling frame. This imbalance may introduce **selection and participation bias**, as female students may have been more likely to respond to a health-related survey, including one focused on ocular symptoms.

 We agree that this skewed gender composition may influence the observed association between female sex and ocular morbidity, and that this finding should be interpreted with caution. Accordingly, the manuscript has been revised to explicitly highlight **female overrepresentation as a major limitation**, and to state that the statistically significant association observed may, in part, be an artifact of the sample composition rather than a definitive biological or behavioral relationship.  

 2. Results: Inconsistencies and Misinterpretation:

 Prevalence Confusion: The Abstract and Results sections present conflicting data. The Abstract states: "64.7% were suffering from ocular morbidities." The Results section (Page 4) states: "202 students (64.7%) were suffering from ocular morbidities currently. Rest of the 110 students (35.3%) had ocular morbidities in the past." This implies a 100% lifetime prevalence, which is confusing and biologically improbable. The text must clearly differentiate between point prevalence (current) and lifetime prevalence, and the logic of this categorization should be explained.

**Response- **We thank the reviewer for identifying this important issue regarding prevalence reporting. We agree that the earlier wording was ambiguous and could be misinterpreted. The manuscript has been revised to clearly distinguish between **current (point) prevalence** and **absence of current ocular morbidity. **The prevalence reported in the Abstract, methods and Results now consistently reflects **point prevalence**, defined as the proportion of students reporting a current ocular morbidity at the time of data collection.

 Critical Error in Conclusion: The Abstract and Conclusion state that "appropriate posture while reading" is associated with ocular morbidity. This is a direct misinterpretation of the data in Table 3, which clearly shows that "Wrong" posture is significantly associated with morbidity (P < 0.0001). The manuscript must be corrected to state that inappropriate posture is a risk factor. This error fundamentally changes the public health message of the study.

**Response-** We thank the reviewer for identifying this critical interpretative error. We agree that the wording in the Abstract and Conclusion was incorrect and did not accurately reflect the findings presented in Table 3. The statistical analysis demonstrates a significant association between **inappropriate (“wrong”) reading posture** and the presence of ocular morbidity (P < 0.0001), and not with appropriate posture.

 Accordingly, the manuscript has been revised to clearly state that **inappropriate reading posture is associated with ocular morbidity**. This correction has been applied consistently across the Abstract, Results, Discussion, and Conclusion to ensure accurate interpretation and alignment with the data.

 Abstract and conclusion has been rephrased - Inappropriate reading posture was significantly associated with ocular morbidity among medical students.

 Conclusion- This study found that refractive errors were the most prevalent ocular morbidities among medical students, with myopia being the most common. Several factors were **associated** with the presence of ocular morbidity, including family history of ocular conditions, female sex, inappropriate reading posture, prolonged screen time, close device viewing distance, and multivitamin supplementation.

 Ocular morbidities were perceived by students to have a negative impact on both lifestyle and academic activities, particularly through reduced participation in sports and recreational activities, perceived limitations in career aspirations requiring optimal visual acuity (such as the Armed Forces), and difficulty sustaining prolonged periods of study. These findings highlight the need for early identification of ocular morbidities, promotion of ergonomic practices and visual hygiene, and implementation of preventive and health education interventions targeted at medical students.

 3. Data Analysis and Presentation:

  Unjustified Data Dichotomization: Figure 1 presents screen time in multiple categories (1-2 hrs, 3-4 hrs, etc.). However, for analysis in Table 3, screen time is dichotomized into >5 hrs vs. <5 hrs. The rationale for choosing this specific cutoff point is not provided. this arbitrary dichotomization loses information and should be justified,or analysis using the original categories should be considered.

**Response-** We thank the reviewer for this important methodological observation. We agree that dichotomization of a multi-category variable can lead to loss of information if not adequately justified.

 In this study, screen time was dichotomized at a cutoff of **≥5 hours per day** versus **<5 hours per day** for analytical purposes based on **prior evidence and public health relevance**, as prolonged daily screen exposure beyond 4–5 hours has been consistently associated with increased risk of digital eye strain and asthenopic symptoms among students and young adults. This cutoff was therefore chosen to distinguish between comparatively lower and higher risk exposure groups.

 To improve transparency, we have now explicitly stated the rationale for this cutoff in the Methods section. In addition, we have revised the Results and Discussion to clarify that this categorization and mentioned in limitations.

  · Confusing Table 3 Format: Table 3 is poorly constructed and difficult to interpret. The column headers are inconsistent. For risk factors 1-7, the columns are "Yes" and "No," but for factors 8 and 9 (Age of onset, Routine check-ups), the columns shift to "Improved or No change" and "Worsened." This creates confusion about what is being compared. The table should be redesigned for consistency and clarity, ideally comparing the presence/absence of ocular morbidity across all risk factor categories.

**Response**- We thank the reviewer for this important observation regarding the presentation of Table 3. We agree that the earlier format was inconsistent and could lead to confusion in interpretation, as different outcome constructs were inadvertently combined within the same table.

 In response, Table 3 has been **redesigned for conceptual and structural consistency**. The revised table now uniformly compares the **presence and absence of current ocular morbidity** across all risk factor categories. Variables related to disease progression or management outcomes (such as age of onset and routine eye check-ups) have been removed from Table 3 and are now presented separately as descriptive findings in result section to avoid mixing distinct analytical constructs.

   Result was rephrased as- Among students with ocular morbidity, earlier age of onset (≤15 years) was more frequently associated with worsening of symptoms compared to later onset (>15 years) ( *p* < 0.0001). Students who had undergone an eye examination within the past six months more commonly reported improvement or no change, whereas worsening was more frequent among those with longer intervals since their last check-up ( *p* < 0.0001).

 Figure 3 (Challenges and Solutions): While informative, this figure is cluttered and difficult to read. It is recommended to split this into two separate, clear lists or a structured table to improve readability.

**Response-** We agree that the original presentation of Figure 3 was visually dense and could impede readability. In response, the figure has been **revised to improve clarity and interpretability while retaining its conceptual value**.

 Specifically, the content has been reorganized into **two clearly demarcated sections within the same figure**, explicitly labeled as *Challenges* and *Solutions*, with simplified text and improved spacing. Redundant wording has been minimized, and the number of items displayed has been streamlined to enhance visual clarity. A concise legend has also been added to clarify that the figure represents **self-reported challenges and coping strategies** and does not imply hierarchy or temporal sequence.

 4. Underdeveloped Limitations Section:

    · The current limitations mention only "a small sample size" and "an online questionnaire." This is insufficient. The section must be expanded to explicitly discuss:

      · The non-probability sampling method and high risk of selection bias.

      · The severe gender imbalance in the sample and its implications.

      · Reliance on self-reported data for both morbidity and risk factors (susceptible to recall and social desirability bias).

      · The cross-sectional design, which precludes any causal inferences. The language throughout the manuscript should be tempered from "causing" or "effects" to "associated with."

**Response -** We thank the reviewer for this important and constructive comment. We agree that the original Limitations section was insufficiently detailed. In response, the Limitations section has been **substantially expanded** to explicitly address key methodological constraints, including the sampling approach and associated risk of selection bias, the marked gender imbalance among respondents, reliance on self-reported data, and the inherent limitations of a cross-sectional design.

 Minor Issues- 

 Title: The subtitle "An academic collateral" is unclear. Suggest a more standard and descriptive subtitle such as "A cross-sectional study."

**Response-** Evaluation of risk factors for ocular morbidities and their impact on the lives of medical students: A cross-sectional study unveiling the academic collateral

 Introduction: The rationale for specifically studying medical students (vs. other university students) could be strengthened by emphasizing unique stressors like prolonged microscope use, vast reading loads, and night-duty schedules.

**Response-** We thank the reviewer for this valuable suggestion. We agree that the rationale for focusing specifically on medical students can be strengthened. Accordingly, the Introduction has been revised to explicitly highlight the **unique academic and occupational visual demands** faced by medical students, including prolonged microscope use during practical sessions, extensive reading requirements, sustained digital screen exposure, and irregular schedules with late-night studying and clinical duties. These factors collectively distinguish medical students from other university populations and provide a strong justification for their focused evaluation in relation to ocular morbidities.

 Author List & Roles (Page 2): There is a discrepancy. The author list includes "Swati Shikha," but the "Author roles" section lists "Kimura A" and "Jungla K." These names ("Kimura A," "Jungla K") do not appear in the main author list and are likely errors that need correction to "Swati Shikha" and "Khushboo Juneja."

**Response- **We appreciate the reviewer’s attention to detail, which has helped improve the accuracy and integrity of the manuscript. The list of authors are- Haneen Haneen, Jarina Begum , Syed Irfan Ali, Abhishek Kumar, Swati Shikha, Khushboo Juneja

 Results Text (Page 4): The phrasing "where they were not inability to see the blackboard" appears to be a grammatical error or incomplete sentence.

**Response- **We thank the reviewer for identifying this grammatical and clarity issue. We agree that the phrasing was incorrect and could lead to confusion. The sentence has now been revised for grammatical accuracy and improved clarity. Result is rephrased as- The most common presenting complaint was headache (56%), followed by blurred vision (32%), including difficulty seeing the blackboard, redness, dryness, and watering of eyes (8.2%), and routine eye check-ups (3.8%).

 Discussion: The first sentence states "The majority (57.4%) had myopia," but the Results on Page 4 state the prevalence among those with morbidity is 84.3%. Please verify and use consistent statistics.

**Response- **We thank the reviewer for pointing out this inconsistency. This study describes the spectrum of ocular morbidities among undergraduate medical students and explores associated risk factors and perceived impacts on lifestyle and academic activities. Among the 202 students with current ocular morbidity, refractive errors were predominant, with myopia accounting for 84.3% of cases. Overall, myopia was present in 57.4% of the total study population (n = 312).

 References: Reference #2 appears to be cut off or corrupted: "(POP) Prevalence of Smart Phone Users at Risk...". This needs to be checked and formatted correctly.

**Response: **The reference 2 has been corrected as - Sadagopan AP, Manivel R, Marimuthu A, et al. Prevalence of smart phone users at risk for developing cell phone vision syndrome among college students. *Journal of Psychology & Psychotherapy*. 2017;7:299.

# Reviewer report: Reviewer response for version 1

**DOI:** 10.5256/f1000research.184311.r433460

**Published:** 2025-12-23

**Authors:** Akinsola S Aina

**Affiliations:** 1 Bowen University, Iwo, Nigeria

1. The methodology is too scanty for reproducibility, e.g. Was ocular examination carried out on these study participants at al? if yes, which eye care professional did what? who did refraction? who carried out fundoscopy? what are the other ocular morbidities in them? hyperopia? Astigmatism, allergic conjunctivitis, Dry eye etc.....................

 2. Was the study carried out among the medical students that has ocular complaints only? so give details of sampling technique used.

 3. Was the 3rd objective of impact on lifestyle and academics of medical students assessed at all... If done .... Please explain further.

Is the work clearly and accurately presented and does it cite the current literature?

Partly

If applicable, is the statistical analysis and its interpretation appropriate?

Partly

Are all the source data underlying the results available to ensure full reproducibility?

Yes

Is the study design appropriate and is the work technically sound?

Yes

Are the conclusions drawn adequately supported by the results?

No

Are sufficient details of methods and analysis provided to allow replication by others?

No

Reviewer Expertise:

Ophthalmology(Cornea and Anterior Segment; Medical Retina)

I confirm that I have read this submission and believe that I have an appropriate level of expertise to confirm that it is of an acceptable scientific standard, however I have significant reservations, as outlined above.

# Response

**Published:** 2026-01-02

**Authors:** Jarina Begum

**Affiliations:** Community Medicine, Manipal Academy of Higher Education, Jamshedpur, Jharkhand, India

We, the authors of this article, sincerely thank the reviewer for the detailed and constructive critique of our manuscript titled **“Evaluation of risk factors for ocular morbidities and its impact on the lives of medical students”**. The comments have helped us improve the methodological clarity, transparency, and interpretability of our study. Our point-wise responses are provided below.

 1. The methodology is too scanty for reproducibility, e.g. Was ocular examination carried out on these study participants at al? if yes, which eye care professional did what? who did refraction? who carried out fundoscopy? what are the other ocular morbidities in them? hyperopia? Astigmatism, allergic conjunctivitis, Dry eye etc.....................

**Response-** We appreciate this important observation. We would like to clarify that this was a questionnaire-based cross-sectional study, and no direct clinical ocular examinations (such as refraction, fundoscopy, or slit-lamp examination) were conducted as part of the study protocol.

 Information on ocular morbidities (including myopia, hypermetropia, astigmatism, strabismus, glaucoma, dry eye, allergic conjunctivitis, and other self-reported conditions) was collected through self-reported history of prior diagnosis and prescriptions. To improve accuracy, reported morbidity details were subsequently verified through structured telephonic interviews and cross-checked with available medical records or prescriptions, where feasible, by subject experts.

 We have now explicitly clarified this in the Methodology section to avoid ambiguity and to ensure reproducibility.

 Revised statement regarding this under the data collection sub-section of the Methodology section of the article is as follows-

 This was a questionnaire-based cross-sectional study, and no direct clinical ocular examinations (including refraction, slit-lamp examination, or fundoscopy) were performed as part of the study protocol. Information on ocular morbidities—such as refractive errors (myopia, hypermetropia, astigmatism), strabismus, glaucoma, dry eye disease, allergic conjunctivitis, and other reported ocular conditions—was obtained through self-reported history of prior diagnosis, use of corrective measures, and available prescriptions.

 For participants reporting ocular morbidities, additional details were collected through structured telephonic interviews. Where feasible, these self-reported details were cross-verified with available medical records or prescriptions and reviewed by subject experts prior to inclusion in the final analysis, to enhance data accuracy and reproducibility.

 2. Was the study carried out among the medical students that has ocular complaints only? so give details of sampling technique used.

**Response: **Thank you for highlighting this point. The study was not restricted to students with ocular complaints only. A complete enumeration sampling technique was employed, wherein all eligible undergraduate medical students were invited to participate irrespective of their ocular health status until the required sample size was attained.

 The methodology has been rephrased as- The study was conducted among undergraduate MBBS students enrolled across all academic phases in two medical colleges in the study area, with an approximate total student population of 800. The study was not restricted to students with ocular complaints.

 A complete enumeration sampling approach was adopted, whereby all eligible medical students were invited to participate irrespective of their ocular health status, until the required sample size of 312 was achieved. This approach ensured representation across all academic phases and allowed estimation of the prevalence and pattern of ocular morbidities within the study population.

 Undergraduate medical students aged 18–26 years who were currently enrolled in the MBBS course and provided informed consent were included. Students who submitted incomplete or partially filled questionnaires or were unable to provide reliable information regarding their ocular health status were excluded from the analysis.

 3. Was the 3rd objective of impact on lifestyle and academics of medical students assessed at all... If done .... Please explain further.

**Response:** Yes, the third objective was assessed. To improve clarity, we have now expanded the Methods section to explicitly describe how lifestyle and academic impact were assessed, and the Discussion has been reframed to reflect perceived impact rather than objective academic performance.

 The methodology was rephrased under the sub-section of study tool as- The impact of ocular morbidities on lifestyle and academic activities was assessed using a dedicated section of the semi-structured questionnaire. Academic impact was evaluated through self-reported difficulties related to educational activities, including challenges in prolonged reading, extended screen use, viewing classroom teaching aids (such as blackboard or projected slides), and use of microscopes during practical sessions.

 Lifestyle impact was assessed by documenting self-reported restrictions in routine and recreational activities, including participation in sports, swimming, night-time driving, and perceived limitations related to future career aspirations (e.g., eligibility for armed forces or other visually demanding professions).

 In addition to closed-ended items, open-ended questions were included to capture participants’ perceived challenges related to ocular morbidity and the coping strategies or solutions adopted. Responses to open-ended questions were analysed using thematic analysis to identify recurring patterns and themes.

 The added paragraph in the discussion section is- It is important to note that the impact described in this study reflects **students’ perceptions and experiences**, rather than objectively measured academic performance or functional outcomes. The inclusion of open-ended responses allowed students to articulate individualized challenges and adaptive strategies, highlighting the broader psychosocial and lifestyle implications of ocular morbidity beyond clinical diagnosis alone.

 We hope that these revisions adequately address the reviewers’ queries and facilitate progression of the manuscript through the peer review process toward approval.
